# Cellular immunotherapy for refractory hematological malignancies

**DOI:** 10.1186/1479-5876-11-150

**Published:** 2013-06-19

**Authors:** John L Reagan, Loren D Fast, Howard Safran, Martha Nevola, Eric S Winer, Jorge J Castillo, James N Butera, Matthew I Quesenberry, Carolyn T Young, Peter J Quesenberry

**Affiliations:** 1 Division of Hematology/Oncology, Rhode Island Hospital/The Miriam Hospital, The Warren Alpert Medical School of Brown University, Providence, USA; 2 Department of Pathology, Rhode Island Blood Center, The Warren Alpert Medical School of Brown University, Providence, USA

**Keywords:** Acute myeloid leukemia, Immunotherapy, Hematological malignancies, Non-Hodgkin lymphoma, Cellular therapy, Cytokine release syndrome

## Abstract

**Background:**

Acute myeloid leukemia (AML) and other aggressive refractory hematological malignancies unresponsive to upfront therapy remain difficult conditions to treat. Often, the focus of therapy is centered on achieving complete remission of disease in order to proceed with a consolidative stem cell transplant. At issue with this paradigm is the multitude of patients who are unable to achieve complete remission with standard chemotherapeutic options. A major benefit of transplantation is the graft versus tumor effect that follows successful engraftment. However, with this graft versus tumor effect comes the risk of graft versus host disease. Therefore, alternative treatment options that utilize immunotherapy while minimizing toxicity are warranted. Herein, we propose a novel treatment protocol in which haploidentical peripheral blood stem cells are infused into patients with refractory hematological malignancies. The end goal of cellular therapy is not engraftment but instead is the purposeful rejection of donor cells so as to elicit a potent immune reaction that appears to break host tumor tolerance.

**Methods/design:**

The trial is a FDA and institutional Rhode Island Hospital/The Miriam Hospital IRB approved Phase I/II study to determine the efficacy and safety of haploidentical peripheral blood cell infusions into patients with refractory hematological malignancies. The primary objective is the overall response rate while secondary objectives will assess the degree and duration of response as well as safety considerations. Patients with refractory acute leukemias and aggressive lymphomas over the age of 18 are eligible. Donors will be selected amongst family members. Full HLA typing of patients and donors will occur as will chimerism assessments. 1-2x10^8^ CD3+ cells/kilogram will be infused on Day 0 without preconditioning. Patients will be monitored for their response to therapy, in particular for the development of a cytokine release syndrome (CRS) that has been previously described. Blood samples will be taken at the onset, during, and following the cessation of CRS so as to study effector cells, cytokine/chemokine release patterns, and extracellular vesicle populations. Initially, six patients will be enrolled on study to determine safety. Provided the treatment is deemed safe, a total of 25 patients will be enrolled to determine efficacy.

**Discussion:**

Cellular Immunotherapy for Refractory Hematological Malignancies provides a novel treatment for patients with relapsed/refractory acute leukemia or aggressive lymphoma. We believe this therapy offers the immunological benefit of bone marrow transplantation without the deleterious effects of myeloablative conditioning regimens and minus the risk of GVHD. Laboratory correlative studies will be performed in conjunction with the clinical trial to determine the underlying mechanism of action. This provides a true bench to bedside approach that should serve to further enrich knowledge of host tumor tolerance and mechanisms by which this may be overcome.

**Trial registration:**

NCT01685606.

## Background

Despite recent improvements in the care of patients with acute myeloid leukemia (AML), the overall prognosis remains dismal with a 5-year relative survival rate of slightly over 20% [1]. Typically, AML patients eligible for curative therapy are treated with high dose chemotherapy involving an anthracycline. If remission is achieved, the majority of patients undergo allogeneic stem cell transplantation (allo-SCT) for definitive curative treatment if, based on age and other medical co-morbidities, they are deemed clinically eligible [2]. Much of the therapeutic effect of stem cell transplantation arises from a graft versus leukemia (GVL) response. Unfortunately, GVL is associated with potential complications such as treatment-related morbidity/mortality from conditioning chemotherapy regimens as well as graft-versus-host disease (GVHD). Furthermore, even after complete response and transplantation there is a 30% probability of disease relapse, and patients who experience a relapse have a 2-year overall survival (OS) of 10-14% [3].

Other hematological malignancies such as acute lymphocytic leukemia (ALL) employ allo-SCT as upfront therapy in poor prognostic settings such as Philadelphia chromosome positive ALL while others, like aggressive non-Hodgkin lymphoma (NHL), utilize allo-SCT in cases of relapsed disease. As is the case in AML, patients with ALL and NHL requiring allo-SCT have poor 5 year OS of 30-38% [4-6] and 24-27% [7,8], respectively. Similar to AML, allo-SCT of relapsed ALL and NHL is limited by treatment related morbidity/mortality [7-9]. Hence, additional therapeutic modalities for acute leukemia and aggressive lymphomas are needed. Herein, we propose an alternative mechanism for cell-directed immunotherapy in relapsed and refractory hematological malignancies that does not require high-dose chemotherapy for conditioning. In our protocol, termed cellular immunotherapy, the goal of cell infusion is not engraftment but instead is the purposeful rejection of donor cells so as to elicit a complex cytokine storm that, we believe, breaks host tumor tolerance.

### Laboratory origins of cellular immune therapy

The origins of cellular immunotherapy stem from early work describing engraftment without myeloablation. Based on the previous work of others, we initially studied the capacity of murine marrow cells to engraft into non-irradiated mice [10]. From the experimental data obtained, we concluded that donor cell engraftment is not only possible without myeloablation but is also quantitative in nature by virtue of competition between host and donor cells [11]. Greater numbers of donor cells infused results in higher levels of engraftment. However, in the clinical setting non-myeloablative engraftment is not possible due to the high number of donor cells that would be required. Therefore, we next examined levels of syngeneic murine engraftment with minimal myeloablation. Utilizing small doses of radiation (50-100 cGy) we demonstrated significant engraftment [12]. The thought behind efficacy in minimal myeloablation was that small radiation doses were stem cell toxic but not myelotoxic. Initial attempts by our group to translate these findings to the allogeneic setting were unsuccessful secondary to immune barriers. Eventually, however, durable non-myeloablative engraftment was possible through anti-CD40 ligand antibody co-stimulatory blockade in the setting of multiple stem cell infusions [13].

### Clinical origins of cellular immune therapy

#### Infusion of HLA identical pheresed cells

The above murine studies became the foundation of a clinical approach for patients with refractory leukemia and lymphoma. Initially, we infused graded doses of human leukocyte antigen (HLA) identical allogeneic T-cells into eleven minimally myeloablated (100 cGy total body irradiation) patients with refractory hematological malignancies in an attempt to achieve durable engraftment. Nine patients achieved mixed or complete chimerism. Four patients attained a complete response (CR), two of which were of long duration. CR was achieved in the single patient treated for refractory AML, in two patients with NHL and in one patient with multiple myeloma (MM). Five patients developed significant acute GVHD, resulting in one death. Of the patients achieving a CR, three developed 100% chimerism. The fourth patient in CR had only a transient 5% chimerism for one week but interestingly had a sustained CR of over 42 months. Another patient with chronic lymphocytic leukemia (CLL) showed a 75% reduction in lymphadenopathy despite no evidence for chimerism thereby suggesting that the cellular infusion may have activated the patient’s own immune system against their hematologic malignancy. In the end, we determined the infusion of 1 × 10^8^ T-cells per kg along with a median of 5 × 10^4^ CD34+ cells per kg from non-mobilized HLA identical blood was safe, effective, and warranted further clinical study [14].

#### Infusion of HLA haploidentical pheresed cells

In order to increase the number of patients eligible for cellular infusion, we next evaluated the infusion of haploidentical peripheral blood cells into 26 patients with refractory hematologic malignancies [15]. Granuloctye colony stimulating factor (G-CSF) mobilization was used for collection of peripheral blood cells. Following 100 cGy of total body irradiation (TBI), patients were infused with escalating levels of CD3+ cells. The study recruited thirteen patients with AML, six with NHL, five with MM, one with ALL and one with chronic myeloid leukemia (CML). No responses were seen in the eight patients treated with 1 × 10^6^ or 1 × 10^7^ CD3+ cells per kg. At higher CD3+ dose levels of 1–2 × 10^8^ per kg, objective responses were seen in 14 out of 18 patients. Two of six patients with NHL remained free of disease at 76 months and 82 months, respectively, while two additional NHL patients obtained partial responses (PR). There were 3 durable CR lasting 8, 11 and 31 months, respectively, and 7 transient responses in 13 patients with AML (Table 1). Remarkable, all responses occurred outside of donor chimerism. Serial bone marrow biopsies performed in several patients showed evidence of large tumor reduction and early resumption of normal hematopoiesis.

**Table 1:** **Summary of responses seen in 26 patients with refractory hematological malignancies treated with haploidentical PBSC infusions [**[15]**]**

**Malignancy**	**Complete response**	**Partial response**	**Transient response**	**Total response**
AML	3/13 (23%)	0/13 (0%)	7/13 (54%)	10/13 (77%)
NHL	2/6 (33%)	2/6 (33%)	0/6 (0%)	4/6 (66%)

Toxicities included well-tolerated myelotoxicity. An immediate post-infusion immunologic syndrome, which we termed “haploimmunostorm”, was observed. This was universally characterized by fever and variably by skin rash, diarrhea, liver dysfunction, effusions, respiratory distress and edema. These signs and symptoms were variable between patients. It only occurred in patients infused with at least 1 × 10^8^ CD3+ cells per kg, began 14 hours after cell infusion, and remitted rapidly with methylprednisolone, 2 mg/kg/day within 6–8 hours of the onset of the haploimmunostorm. Typically, we allowed this syndrome to persist for at least 48 hours as it was hypothesized that this might be a component of the therapeutic response.

Donor chimerism, as determined by short tandem repeat testing in multiplex battery with 1-5% sensitivity, was not seen in most patients. Only two patients developed donor chimerism and both patients died; one patient clearly died of GVHD and the other possibly from GVHD. Hence, we were able to document complete responses, some of which were durable, with cell infusions in the absence of demonstrable engraftment.

Evaluation of serum cytokine levels during the haploimmunostorm revealed elevations of multiple cytokines including interferon γ which in the murine model has been shown to play a role in the host versus tumor response [16,17]. The pattern of cytokine elevations was distinct from the patterns seen with the engraftment syndrome and GVHD. These elevations were felt to be related to the haploimmunostorm manifestations.

### Cytokine release syndrome; other studies

Similar to the description of the haploimmunostorm phenomenon is a cytokine release syndrome (CRS) that has been observed in the chimeric antigen receptor (CAR) modified T-cells studied in CLL patients by the University of Pennsylvania group [18]. In their work, the cytokines IFN γ and IL-6 are elevated in patients whose CLL responds to infusion of modified CAR T-cells while those patients who exhibit no CRS show no response to this therapy. These initial findings in CLL have been reproduced in patients with ALL [19]. The clinical findings of hypotension and hypoxia are also similar to our experiences with haploimmunostorm, as is the use of steroids to treat these symptoms. More recently, they have shown blockade of IL-6 with the monoclonal antibody tociluzimab is able to mitigate the side effects of cytokine release syndrome without dampening the anti-tumor activity [20].

### Nonengraftment haploidentical cellular therapy; other studies

The clinical efficacy of cellular therapy has been replicated by Guo and colleagues in a study of patients ≥ 60 with AML [21]. Here, they randomized patients to either receive chemotherapy alone or chemotherapy with haploidentical G-CSF mobilized peripheral blood stem cells (PBSC). In the chemotherapy alone group, the CR rate was 43% while the CR rate in the chemotherapy and PBSC group was 80%. Furthermore, the 2-year progression free survival (PFS) with chemotherapy alone was 10% in contrast to a 2-year PFS of 39% in the chemotherapy and PBSC cohort. These results show clear activity for cellular therapy in patient population with a notoriously dismal prognosis. A subsequent study performed by Guo et al. infused HLA-mismatched donor G-CSF mobilized PBSC following 3 cycles of cytarabine for AML patients who attained CR after induction chemotherapy. In total, 101 low- and intermediate-risk AML patients were included in this study. In the low-risk group, the 6-year leukemia free survival (LFS) rate was 84% while the OS was 89%. In the intermediate-risk group the LFS was 59% and the OS was 65% [22]. These are remarkable responses for patients with AML in whom the average 5-year OS is 55% and 24% in similar risk groups [23]. Interestingly, this same group reported a benefit of donor cell infusions with chemotherapy for the treatment of myelodsyplastic syndrome compared to chemotherapy alone [24].

### Study rationale

The infusion of 1–2 × 10^8^ CD3+ haploidentical cells per kg into minimally irradiated patients with refractory lymphoma or leukemia results in dramatic and sometimes durable responses in the absence of engraftment [15,21,22]. In this setting, the patients developed a unique CRS characterized by fever, diarrhea, liver function abnormalities, skin rash and pulmonary symptoms. This suggests the activity in haploidentical blood cell infusion is likely due to activation of the recipient’s immune system against leukemia/lymphoma and not due to graft versus tumor. This response may be related to CRS development as described by our group [15] and others [18,20].

In order to increase the host immune response to haploidentical cellular infusion, no pre-infusion irradiation or chemotherapy will be administered. Furthermore, as G-CSF would be administered to healthy volunteers the unclear benefit of the addition of this cytokine is offset by the potential side effects such as headache, fever, and bone pain. Moreover, G-CSF mobilization serves to shift the response from a T_H_1 to T_H_2 through the increased production of T-regulatory cells thereby potential decreasing the immune response [25]. Therefore, in this study, haploidentical cells will be collected directly from the donor and infused into the patient. The end goal is purposeful rejection of donor cells by the host immune system which, we postulate, results in breakage of host tumor tolerance. The underlying mechanism behind this phenomenon has not been fully elucidated but is thought to involve a complex interaction between interferon γ, CD8+ T-cells, NK cells, and antigen presenting cells. Laboratory correlative studies to determine the mechanism of action will be conducted alongside the clinical trial.

## Methods/design

The current trial is a single-arm phase I/II non-randomized study designed to evaluate the safety and efficacy of haploidentical cellular infusion in patients with refractory acute leukemia and aggressive lymphoma. Additional laboratory correlative studies will be performed in conjunction with the clinical trial to determine an underlying mechanism of action.

### Primary and secondary objectives

The primary objective is to assess the overall response rate of cellular immune therapy with HLA-haploidentical peripheral blood pheresed cells in patients with relapsed/refractory hematological malignancies.

Secondary objectives will more accurately describe the clinical effect by assessing the time-to-progression, PFS and OS for patients with relapsed/refractory hematologic malignances following HLA-haploidentical cellular therapy. An additional secondary objective is to evaluate the rate of dose-limiting toxicities of HLA-haploidentical peripheral blood pheresed cellular infusions.

### Exploratory objectives

These objectives will serve to decipher the underlying process by which cellular therapy results in clinical response. We will evaluate *in vitro* mixed lymphocyte assays of donor and recipient cells to determine if *in vitro* stimulation and cytolytic activity corresponds to clinical efficacy. Furthermore, samples will be taken prior to, during, and after the onset of the cytokine release syndrome in order to determine cytokine release profiles, effector cell populations, and extracellular vesicle release.

### Study design

All patients over the age of 18 with relapsed aggressive lymphoma or acute myeloid/lymphoid leukemia with at least one prior therapy and no curative options are eligible (Table 2). HLA-haploidentical donors over the age of 18 whom are healthy and meet criteria of blood donation are eligible (Table 3).

**Table 2:** Criteria for recipient (patient) enrollment

**Recipient eligibility criteria**	
Inclusion Criteria	Age ≥ 18
	Histologic confirmation of the following leukemias/lymphomas:
	• Mantle cell lymphoma with Ki-67>30%
	• Diffuse Large Cell Lymphoma
	• Burkitts Lymphoma
	• Systemic T Cell Lymphomas
	• Acute Myeloid Leukemia
	• Acute Lymphoblastic Leukemia
	Recurrence or progression of disease after at least 1 prior standard treatment
	Progression of disease within 6 months of last treatment
	No available curative treatment option
	≥ 4-weeks since prior chemotherapy or radiation (Exception: Hydroxyurea may be utilized up to 48 hours prior to treatment)
	Life expectancy of 2 months at treatment initiation
	≥ 6 months post autologous stem cell transplant
	DLCO ≥ 40% with no symptomatic pulmonary disease.
	LVEF ≥ 40% by MUGA or echocardiogram.
	Creatinine ≤ 2.0 mg/dl, Total bilirubin <1.5x the upper limit of normal (ULN), AST < 3x ULN
	Non-pregnant and willing to use appropriate birth control during study period
Exclusion criteria	Previous allogeneic stem cell transplant
	Previous purine analog (fludarabine, pentostatin, 2-CDA) or alemtuzumab within 1 year of entering the study
	CML, CLL, multiple myeloma, and indolent lymphoma (follicular lymphoma, marginal zone lymphoma)
	HLA antibodies to donor HLA type
	HIV-1 or 2 positive
	Oxygen dependant COPD
	Failure to demonstrate adequate compliance with medical therapy and follow-up
	Significant medical or psychiatric illness that would impair the ability to participate in protocol therapy
	Active systemic infection

**Table 3:** Criteria for haploidentical PBSC donation

**Donor eligibility criteria**	
Inclusion criteria	Biological family members
	3/6 HLA match (using loci A, B, DR)
	18 years of age
	No malignancy in the past 5 years except for non-melanoma skin cancers
	Normal CBC
	β-HCG urine or serum negative if donor is of childbearing age
	Adequate venous access so leukapheresis can be performed via standard peripheral IV
Exclusion criteria	HIV-1 or 2, syphilis, hepatitis B or C, HTLV 1 or 2, CMV, Chagas, and West Nile Virus positive
	Symptomatic congestive heart failure (CHF)
	Oxygen dependant chronic obstructive lung disease (COPD)
	Cirrhosis or active liver disease
	History of any lymphoid, myeloid or other non-solid malignancy
	History of transplantation

Selected donors will undergo leukapheresis. The final product will be analyzed for CD3+ and CD34+ content via flow cytometry. The product will be administered unprocessed on day 0 (same day as leukapheresis) with 1–2 × 10^8^ CD3+ cells per kg irrespective of the number of CD34+. No specific viral or bacterial prophylaxis is required. The infusion of HLA-haploidentical peripheral blood cells must be initiated within 8 hours of product collection and completed within 24 hours. Acetaminophen 650 mg PO and diphenhydramine 50 mg IV will be administered 30 minutes prior to haploidentical infusion. During the infusion, patient will be monitored for blood pressure, temperature and oxygen saturation. This monitoring will continue for 2 hours after infusion at the following time points; every 15 minutes the first hour post infusion and every 30 minutes the second hour post infusion.

The infusion will be stopped should the patient develop grade 3 or 4 infusion-related reactions. For grade 2 infusion-related reactions the following protocol will be followed:

1^st^ occurrence of Grade 2 infusion related reaction

1. Infusion placed on hold

2. Acetaminophen 650 mg PO × 1

3. Diphenhydramine 50 mg IV × 1

4. Ranitidine 50 mg IV ×1

5. Infusion restarted following improvement in symptoms to Grade 1 or less

2^nd^ occurrence of Grade 2 infusion related reaction

1. Infusion placed on hold

2. Methylprednisolone 100 mg IV × 1

3. Infusion restarted following improvement in symptoms to Grade 1 or less

### Toxicities/Safety

**Figure 1:**
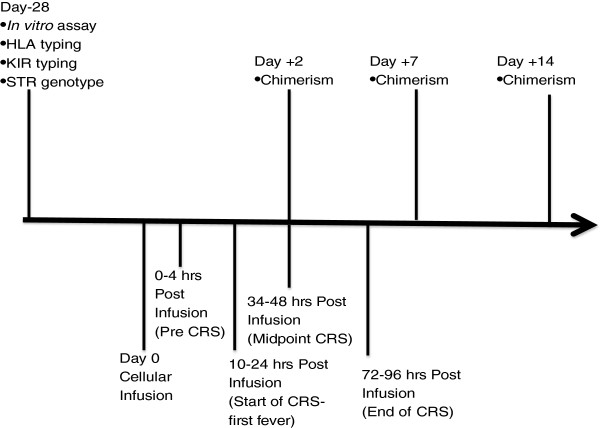
Schematic depicting key time points for blood sample accrual for translational studies to be incorporated into the Cellular Therapy Protocol (HLA= Human leukocyte antigen, KIR= Killer-cell immunoglobulin-like receptor, STR= short tandem repeat, CRS= Cyokine release syndrome).

Patients will be monitored for cell infusion syndrome and cytokine release syndrome. Patients will be given methylprednisolone for Grade 3 or 4 Toxicities based on NCI CTC version 4.0 criteria. Furthermore, percent chimerism will be assessed using short tandem repeats 2 days post cellular infusion, 14 post cellular infusion, and every 14 days thereafter as long as chimerism persists (Figure 1). Lack of chimerism will obviate the risk of GVHD. After 6 patients have been followed for a minimum of 2 months after cellular infusion, the Brown University Oncology Research Group (BrUOG) will review safety data prior to reopening accrual. Data from the safety review will be shared with the FDA.

Adverse events will be scored according to the NCI CTCAE version 4 criteria. Dose limiting toxicity (DLT) will be defined as any of the following treatment related events:

• Grade 3 graft versus host disease lasting > 7 days

• Grade 4 graft versus host disease of any duration

• Grade 3 infusion related symptoms lasting > 7 days

• Grade 4 infusion related symptoms of any duration

In the safety run-in for the first 6 patients, if 2 or less of 6 patients have a dose limiting toxicity then accrual will be allowed to extend to a total of 25 patients. If 3 or more of 6 patients have a DLT, or there is one grade 5 treatment-related adverse events, protocol accrual will be suspended. If this circumstance occurs, the BrUOG data safety monitoring group will review the adverse event data and make appropriate recommendations to the BrUOG scientific advisory board and the FDA about the study.

The safety evaluation of all 25 evaluable patients is a major objective of this study. A 35% rate of treatment-related adverse events from cellular infusion will be considered unacceptable.

### Response criteria

Criteria for AML and ALL: Includes complete response (CR), partial response (PR), transient response, treatment failure, treatment failure due to patient death, and relapse [26] (Table 4). Response Criteria for Lymphoma: Includes CR, PR, stable disease (SD), relapsed disease or progressive disease (PD) [27] (Table 4).

**Table 4:** **Response criteria for cellular immunotherapy in both leukemia and lymphoma [**[26]**,**[27]**]**

	**AML/ALL**	**Lymphoma**
Complete Remission (CR)	• No leukemic blasts in PB	Nodal
	• No extramedullary leukemia	• If PET positive prior to treatment then any mass provided PET negative. If PET negative or variably positive then nodal regression to normal size on CT
	• BM cellularity > 20% without Auer rods and <5% blasts	
	• ANC > 1.0 × 10^9^/L	Spleen/Liver
	• Platelet count > 100x10^9^/L.	• Not palpable, resolution of nodules
		Bone Marrow
		• Morphological clearance or if morphology equivocal then IHC normalization
Partial Remission (PR)	• All criteria for CR except bone marrow may have 5-20% blasts	Nodal
		• ≥ 50% decrease in the SPD of up to 6
		• largest masses
		Spleen/Liver
		• ≥ 50% decrease in SPD of nodules if multiple nodules, ≥ 50% in size of the transverse diameter of a single nodule if solitary mass
		Bone Marrow
		• No new bone marrow involvement
Transient Response (TR)	• Loss of PB blasts	N/A
	• >50% reduction in BM blasts	
Stable Disease (SD)	N/A	• If PET positive pretreatment, then PET positive post treatment at previous sites. If PET negative pretreatment then no CT change in size of previous lesions.

### Statistical considerations

As noted above, the safety of cellular therapy administration in the first six patients will be reviewed by the FDA and the BrUOG Data Safety Monitoring Board (DSMB). These reviews will be shared with our institutional IRB. If the treatment appears safe, as determined by the FDA and the BrUOG DSMB with institutional IRB agreement, the protocol may be reopened to treat a total of 25 patients. A sample size of 25 patients will differentiate between a 10% level of activity and a 30% level of activity. Specifically, the hypothesis to be tested is:

H_0:_ p< 0.1 versus H_1_: p> 0.3

A Simon two-stage design will be used in this study. The first 15 evaluable patients will be assessed for activity. The trial will be terminated early if activity is observed in 0 or 1 of these 15 patients, and it will be concluded that the true activity rate is unlikely to be > 10%. If activity is demonstrated in at least 2 patients, accrual will continue until a total of 25 evaluable patients are enrolled. If activity is observed in 5 or fewer of 25 patients, the null hypothesis will be accepted and it will be concluded that there is not sufficient activity to merit further investigation of the regimen. Otherwise, it will be concluded that the treatment regimen has sufficient activity to warrant further investigation.

The characteristics of this study design are as follows:

This design yields a type I error rate of <0.05 (α=0.03) and power of 80% when the true response rate is 30%.

Overall survival, time to progression, and progression free survival will be determined by the Kaplan Meier method (from the time of study enrollment).

## Translational endpoints

### Prospective studies for evaluation of donor and patient alloreactivity

Within our center we have created an *in vitro* study in which inactivated randomly selected mismatched donor cells are mixed with CD3+ cells from leukemia patients. Stimulated CD3+ patient cells are then placed on ^51^Cr labeled leukemic blasts with cytolytic activity measured by ^51^Cr release. Preliminary results obtained thus far show cytolytic anti-leukemic activity in approximately half of the stimulated patient CD3+ cells [28]. Because these results are about the same frequency as the responses to cellular immunotherapy, it raises the question of whether this *in vitro* assay would be predictive of *in vivo* responses using the donor/patient combination to be tested. Further, if CD3+ proliferation and cytolytic activity, as determined by *in vitro* assays, is donor dependant then it may be possible to identify an optimal donor. These *in-vitro* studies will be done in a prospective manner by obtaining additional tubes of blood from patient and donor at the day −28 time point (Figure 1). Blood will also be obtained from other individuals that could have been considered as donors or from completely mismatched normal controls.

CD3+ cells from recipient peripheral blood mononuclear cells (PBMC) will be positively isolated using immunomagnetic particles from Miltenyi Biotec^©^. Mitomycin C treated PBMC from donors (stimulators) will be placed in a mixed lymphocyte culture (MLC) with recipient CD3+ cells (responders). CD33/CD34+ cells from Recipient CD3- fraction will be positively selected with immunomagnetic particles from Miltenyi Biotec to create a blast target population for cytolytic studies. MLC proliferation assays will be measured on day 5. On day 7, MLC cells will be collected. Recipient CD33/34+ blasts will be labeled with ^51^Cr MLC stimulated cells will be co-cultured with ^51^Cr labeled blasts for cytolytic assays. The ability to induce the patient’s CD3+ cells to generate anti-leukemic effector cells using the different stimulator cells will be compared to the clinical results subsequently seen in the patient.

### Identification of functional effector cells after cellular infusion

Blood samples will be taken frequently during the first few days after cellular infusion (Figure 1). Blood will be centrifuged with the plasma collected from these samples and aliquots frozen down for future analysis. PBMC will be isolated from the cell pellet using Ficoll-Hypaque discontinuous centrifugation. An aliquot of PBMC will be frozen down for killer-cell immunglobulin like receptor (KIR) expression using Luminex based typing (Gen-Probe) [29]. PBMC analysis will include cell staining for the presence of various subpopulations and their activation status. A panel of anti-HLA antibodies coupled with AlexaFluor 488 that has been shown to be useful for detecting microchimerism during pregnancy in 90% of individuals tested has been made available [30,31]. These antibodies will be used to distinguish donor and recipient cells. In addition to determining the percentage of donor cells circulating in the blood at these different time points, these antibodies can be combined with antibody staining panels that define different subpopulations (CD3, CD4, CD8, CD56, CD69), as well as cytokine and granzyme production by these cells. If there are sufficient cells, it may be possible to use antibody panel staining with anti-HLA antibodies to sort for donor and/or recipient cells. Once collected, these populations could be tested directly for their ability to lyse leukemic target cells or natural killer target cells such as K562. If there are limited number of cells, PCR will be performed to determine the expression of loci expressing effector molecules.

### Identification of cytokine, chemokine, and granzyme profiles and extracellular vesicle release patterns

Cytokine (IL-2, IL-4, IL-6, IL-10, IL-12, IL-15, IL-18, IL-21, IFN γ), chemokine (MCP, MIP1b, CX3CL1) and granzyme (A, B) levels present in the plasma will be determined using multiplex assays (CBA flex set assays or Luminex assays) using the same blood samples obtained for effector cell analysis (Figure 1). In addition to cytokine profiles, patient plasma samples will be examined for extracellular vesicle content. Vesicles will be isolated by ultracentrifugation and examined for protein and RNA content.

## Discussion

Cellular Immunotherapy for Refractory Hematological Malignancies provides a novel treatment for patients with relapsed and refractory acute leukemia or aggressive lymphoma. We believe this therapy could offer the immunological benefit of bone marrow transplantation (i.e. GVL) without the deleterious effects of myeloablative conditioning regimens and the risk of GVHD. Although the exact mechanism of action behind the clinical efficacy remains to be elucidated, we believe that the potential therapeutic benefit is too great to ignore. Furthermore, laboratory correlative studies will be performed in conjunction with the clinical trial. This provides a true bench-to-bedside approach that should serve to further enrich knowledge of host tumor tolerance and mechanisms by which this may be overcome.

The overriding goal of this phase I/II study is to generate clinical responses with a treatment that is safe and well tolerated. The initial six patients enrolled will be re-evaluated for safety considerations with potential alterations to the protocol made in order to enhance both safety and efficacy. The purposeful rejection phenomenon seen within our study could serve as a platform for additional trials of host-mediated immunotherapy for other malignancies.

## Competing interests

The authors declare that they have no competing interests.

## Authors’ contributions

JLR, HS, ESW, JJC, JNB, MIQ, CTY, and PJQ designed the protocol. LDF and MN developed the *in vitro* assay. JLR, LDF, ESW, JJC, and PJQ wrote the manuscript. All authors reviewed and approved the final draft of the manuscript.

## Authors’ information

JLR was recently awarded a Leukemia and Lymphoma Society Special Fellow in Clinical Research Grant for the above work.
